# Exploring the role of vitamin D in cognitive function: mediation by depression with diabetes modulation in older U.S. adults, a NHANES weighted analysis

**DOI:** 10.3389/fnut.2024.1356071

**Published:** 2024-06-04

**Authors:** Chang Shu, Chenguang Zheng, Xin Du, Da Luo

**Affiliations:** ^1^Tianjin Key Laboratory of Cerebral Vascular and Neurodegenerative Diseases, Tianjin Neurosurgical Institute, Tianjin Huanhu Hospital, Tianjin, China; ^2^Tianjin Key Laboratory of Brain Science and Neural Engineering, Tianjin University, Tianjin, China; ^3^Tianjin Huanhu Hospital, Tianjin, China

**Keywords:** mediation analysis, 25-hydroxyvitamin D3, depression, cognitive function, structural equation modeling

## Abstract

**Background:**

The relationship between vitamin D levels, depressive symptoms, and cognitive function has yet to be definitively understood in the elderly, particularly when considering the impact of chronic diseases. This study focuses on how depression mediates the impact of 25-hydroxyvitamin D3 (25(OH)D3) on cognitive performance in older U.S. adults.

**Methods:**

We analyzed data from 2,745 elderly individuals extracted from the NHANES 2011–2014 cycles, applying weighted processing to account for the complex multi-stage sampling design characteristic of NHANES data. Utilizing weighted data for covariate and model selection, we conducted mediation analyses on both the overall population and subgroup data. Significant mediation pathways were validated using a stratified weighted bootstrap approach. For significant subgroup pathways, we explored interactive mechanisms through interactive mediation analysis.

**Results:**

Mediation analyses, thoroughly accounting for the impact of chronic conditions, revealed significant pathways in both the weighted overall population and the weighted diabetes subgroup. After 1,000 stratified weighted bootstrap replications, the proportion of mediation effects were 10.6% [0.040, 0.268] and 20.9% [0.075, 0.663], respectively. Interactive mediation analysis for diabetes indicated that the interaction between diabetes and depression was not significant in the direct pathway (estimates = 0.050, *p* = 0.113) but was significant in the mediation pathway, yielding the largest effect size compared to other covariates (estimates = 0.981, *p* < 0.001).

**Conclusion:**

This study highlights the mediating role of depression in the relationship between vitamin D levels and cognitive function in the elderly, particularly emphasizing diabetes as a key moderator. Our findings suggest targeted interventions addressing both vitamin D sufficiency and depression could significantly benefit cognitive health, especially in diabetic individuals.

## 1 Introduction

Cognitive dysfunction, increasingly prevalent in geriatric populations, not only escalates healthcare burdens but also severely diminishes their quality of life (1). Vitamin D, a fat-soluble steroid, has been demonstrated to have profound effects on physical and mental health due to its significant roles in the cardiovascular, endocrine, and nervous systems. The relationship between vitamin D levels and depression is supported by both the presence of vitamin D-related components in brain tissue and the plausible biological mechanisms through which vitamin D influences neurological functions (2, 3). These mechanisms include the regulation of neurotransmitter synthesis, which is crucial for neurological functions stabilization, and the modulation of inflammatory responses, which have been implicated in the pathophysiology of depression (2, 3). Two follow-up cohort studies have found that vitamin D deficiency is a risk factor for depression in the elderly (4, 5). Additionally, existing research has established that the relationship between depression and cognitive function is unidirectional, with depression occurring prior to the onset of cognitive impairments and serving as a longitudinal risk factor for cognitive decline (6, 7). Therefore, we posit that there may exist a pathway in elderly individuals where vitamin D influences cognitive function through the mediation of depression. This hypothesis highlights the importance of considering the interplay between nutritional status, mental health, and cognitive health in the aging population, suggesting a sequential and interconnected process that warrants further investigation.

The elderly represent a distinct demographic, often grappling with a greater number of chronic conditions than their younger counterparts. Likewise, various chronic diseases are known to influence the onset of depression and cognitive decline. Evidence indicates that older adults with diabetes are at heightened risk for developing both depression and cognitive dysfunction (8, 9). Clinically significant depression is observed in one out of every four individuals with type 2 diabetes mellitus (10). Evidence indicates that diabetes significantly contributes to cognitive dysfunction and predisposes individuals to cognitive decline, with a 50% increased risk of developing dementia (11). Other common chronic diseases among older adults, such as hypertension, stroke, and heart disease, have also been reported to be associated with the occurrence of depression and cognitive dysfunction (12–17). Without accounting for such covariates as chronic diseases, we might struggle to accurately differentiate between the direct and indirect effects of vitamin D levels on cognition, and to precisely determine the significance of the mediating effect of depression. Neglecting these covariates could obscure the true relationship between vitamin D and cognitive outcomes, as well as the mediating role of depression.

In our research, we extend beyond common demographic and lifestyle factors to particularly emphasize the impact of chronic disease-related covariates on how vitamin D and depression mediate cognitive function. This includes considering the effects of chronic diseases both in mediating pathways and direct pathways. Moreover, we have conducted subgroup mediation effect analyses on chronic disease factors that demonstrated statistical significance in preliminary screenings, using them as grouping variables. All analyses, including variable selection, model fitting, and subgroup analyses, are based on the complex stratified weighted data from the National Health and Nutrition Examination Survey (NHANES), utilizing the official NHANES weights.^1^ This approach ensures our findings reflect the real situation across the U.S. civilian, non-institutionalized population. Our objective is to delineate and validate the pathways through which vitamin D impacts cognitive function by mediating depression, with a special focus on the role of chronic diseases.

## 2 Materials and methods

### 2.1 Study population

Data from the 2011–2014 NHANES cycles are used in our study, as only these cycles include our selected research variables. We excluded all rows with missing values in the key variables of cognitive function, depression, and 25(OH)D3. The remaining dataset, comprising 2,745 individuals aged 60 and over, retained covariates with less than 10% missing values for further analysis. The study population, based on complex multistage probability weighted data, consisted of approximately 46% men and 54% women, with a total weighted sample size of 50,667,046. The median age of the weighted sample was 68 years, with an interquartile range of 63 to 74 years (Supplementary Table S1). This survey design ensures that the sample is representative of the non-institutionalized elderly civilian US population, capturing a broad range of demographic and socioeconomic backgrounds to provide a robust basis for our analysis.

Participants were included based on their willingness and ability to participate in comprehensive health assessments. These assessments were conducted by trained interviewers and healthcare providers, either in participants’ homes or at Mobile Examination Centers (MEC). Ethical approval for the data collection was obtained from the National Center for Health Statistics Ethics Review Board, and informed consent was secured from all participants. Detailed interview protocols and measurement methodologies are described in the NHANES documentation.^2^

### 2.2 Cognitive function assessment

The NHANES 2011–2014 cycles included a comprehensive cognitive assessment battery that consisted of three tests: the Consortium to Establish a Registry for Alzheimer’s Disease (CERAD), the Animal Fluency Test (AF), and the Digit Symbol Substitution Test (DSST). The CERAD evaluated immediate and delayed memory function through a set of learning and recall trials involving 10 unrelated words. The assessment was comprised of three Word Learning Subtests and one Delayed Word Recall test. We aggregated these scores to arrive at a total CERAD score (18). The AF assessed categorical verbal fluency by asking participants to name as many animals as possible within a minute, with a preliminary test involving naming three articles of clothing. The DSST gaged processing speed, sustained attention, and working memory by requiring participants to match symbols and numbers on a paper form within 2 min. To create a composite cognitive function score as our dependent variable, we averaged the z-scores of three standardized tests that collectively assess cognitive function (19). Poor cognitive performance was identified as scores falling in the lowest 25th percentile for the composite score, after accounting for NHANES sampling weights, aligning with previous NHANES-based studies (20).

### 2.3 Depression of patient health questionnaire-9 (PHQ-9)

Depressive symptoms were assessed using the Patient Health Questionnaire-9 (PHQ-9), a widely accepted and validated self-rating scale administered during face-to-face interviews at MEC. The PHQ-9 consists of nine items that evaluate various facets of depression, including anhedonia, depressed mood, sleep disturbance, and suicidal ideation, among others. Respondents rated each item on a scale from 0 to 3, resulting in a total score ranging from 0 to 27, where higher scores indicate more severe depressive symptoms. To achieve analytical consistency with cognitive scores and 25(OH)D3 trends, we transformed the PHQ-9 scores using the formula: Depression Score = 27 − Original Score (actual PHQ-9 score). This transformation aligns the depression scores directionally with cognitive function scores and 25(OH)D3 levels, where higher values consistently represent better outcomes, thereby making the model’s interpretation more straightforward and consistent. We analyzed the depression scores as a continuous mediator variable.

### 2.4 Laboratory measurement of 25(OH)D3

The concentrations of serum 25(OH)D3, 25(OH)D2 and C3-epi-25(OH)D3 were analyzed using high-performance liquid chromatography tandem mass spectrometry (LC–MS/MS) at the National Center for Environmental Health, CDC, Atlanta, GA. Calibration and quality control followed NHANES laboratory procedures. The total serum 25(OH)D level was computed by summing the levels of 25(OH)D3 and 25(OH)D2. We selected 25(OH)D3 as the independent variable for our mediation analysis for several reasons. First, it is the most metabolically stable and abundant form of vitamin D metabolite (21), with its serum concentrations serving as a reliable biomarker for assessing an individual’s Vitamin D status (22). Measurement of 25(OH)D3 thus represents a more accurate estimation of *in vivo* vitamin D status (23). Secondly, in the case of the continuous variable 25(OH)D2, the proportion of truncated values is excessively high (74.1%), which compromises its accuracy and, by extension, affects the accuracy of the combined value of total 25(OH)D.

### 2.5 Covariates

We selected covariates from the NHANES dataset based on their relevance to depression and cognitive function, as determined by literature reviews and expert consultation. These covariates include age, gender, poverty income ratio (PIR), marital status, educational background, Body Mass Index (BMI), waist circumference, smoking status, alcohol consumption, season of exam, and historical chronic diseases such as diabetes, hypertension, heart diseases (including congestive heart failure, coronary heart disease, angina/angina pectoris, and heart attack), emphysema, chronic bronchitis, malignancy, hyperlipidemia, stroke, and sleep disorders. All information was acquired through standardized questioning, physical examinations, and laboratory tests, provided by qualified medical staff.

### 2.6 Statistical analysis

To ensure our statistical analysis accurately represents the complex structure of NHANES, we adapted all our statistical analysis methods to align with its sophisticated, multistage, probability sampling design, explicitly incorporating complex weights to achieve accurate estimates that reflect the U.S. population. The cognitive score was divided into quartiles, with quantile thresholds determined using survey-weighted quantile analysis. We present the descriptive results in two forms: weighted data, to provide estimates that are generalizable to the population level, and unweighted data, which represent the raw data without considering the survey’s stratification and clustering. For categorical variables, we display frequencies and percentages, denoted as *n* (%). Continuous variables are described using median values with the interquartile range, indicated as M (Q1, Q3), for both weighted and unweighted data. Variable selection was conducted using weighted data. For comparisons between categorical variables and outcome variable, we applied an adjusted Chi-squared test, which accounts for the design effect in survey data. This adjustment makes the test appropriate for contingency tables, particularly those with small expected frequencies in cells. For continuous variables, the comparison with outcome variable was carried out using a Wilcoxon rank-sum test tailored for complex survey samples. The variables extracted for our study were those with less than 10% missing values. Missing data were filled using Random Forest Imputation via the missForest package. This technique excels at handling nonlinear relationships between variables and offers a robust, high-accuracy approach for imputing both numerical and categorical data without requiring distributional assumptions (24). For subgroup analysis, we selected age, gender, and chronic disease-related variables as grouping factors, ensuring that each subgroup demonstrated a power value greater than 0.8 on the actual number of data points. This criterion guarantees the statistical robustness and representativeness of our analysis, focusing on variables with sufficient statistical power to detect meaningful differences.

From both overall and subgroup perspectives, we employed survey-weighted generalized linear modeling (GLM) with a Gaussian family to assess the effects of the independent variables on the mediator, the mediator on the outcome variable, and the independent variables on the outcome variable. This initial analysis aimed to determine the statistical significance of these relationships, thereby establishing the necessity for mediation analysis. Furthermore, we assessed the interaction effects between 25(OH)D3 and depression within the cohort in a weighted analysis to mitigate the potential influence of interaction effects. This step is critical in ensuring that the mediation analysis is not confounded by unaccounted interaction between variables.

In our analysis, we employed structural equation modeling (SEM) specifically tailored for the complex, weighted survey design. We integrated key aspects of the survey’s methodology, including the designation of primary sampling units (PSUs) and the careful stratification of the data into distinct layers. Each model took into account the hierarchical structure of the data through nesting, alongside the application of survey weights, which are pivotal in ensuring our analyses accurately represent the broader U.S. civilian, non-institutionalized population. By strategically incorporating chronic disease covariates into the mediation and direct effect paths in varying configurations, we constructed five multifactorial mediation analysis models, each designed to explore different aspects of the relationships among chronic conditions, mediators, and outcomes. We conduct a comprehensive evaluation of several fit indices for SEM, including the comparative fit index (CFI), Tucker-Lewis index (TLI), Akaike information criterion (AIC), Bayesian information criterion (BIC), standardized root mean square residual (SRMR), and sample-size adjusted BIC (SABIC), to select the model with the best fit.

The chosen model is then used to perform mediation analysis on both the weighted total population data and weighted subgroup data. The criteria for determining significant pathways were based on the condition that the *p*-values for direct effects, mediation effects, and total effects were all less than 0.05. For the validation of significant pathways, we developed stratified weighted bootstrap mediation analysis for complex survey data, with an emphasis on maintaining the integrity of the survey’s design, including stratification, clustering, and weighting. This approach meticulously generates 1,000 weighted bootstrap samples, with a unique emphasis on ensuring that each stratum within the data has at least two primary sampling units (PSUs). This requirement is crucial for preserving the representativeness and variability of the survey’s stratified design, thereby ensuring that the integrity of the survey structure is upheld in the analysis. For significant subgroup pathways, we adopt the approach of incorporating depression interaction terms into both the mediation and direct paths. This method allows us to analyze the moderating mechanism of the subgroup on depression. Finally, as a sensitivity analysis, we applied the same mediation analysis model to unweighted NHANES data to examine the stability of significant pathways.

All statistical analyses were completed by R 4.2.3 software and the results were considered statistically significant when the bilateral *p*-value <0.05.

## 3 Results

### 3.1 Baseline characteristics of the study population

Table 1 presents the baseline characteristics of the study population, with statistical descriptions provided for both data adjusted using NHANES’s complex survey weights and for unweighted data from the 2011–2014 NHANES dataset. The medians for continuous variables and the proportions for categorical variables differ between the weighted and unweighted data, underscoring the impact of the NHANES complex weighting. Nevertheless, the directional trends across both the weighted and unweighted data remain similar with respect to the association with cognitive function categories. Individuals with conditions such as hypertension, diabetes, heart disease, and stroke are more prevalent in the cohort with poor cognitive function than in those with better cognitive function.

**Table 1:** Characteristics of adults aged 60 and over, stratified by cognitive function category in the 2011–2014 NHANES.

Characteristic	Weighted data (*N* = 50,667,046)		Unweighted data (*N* = 2,745)	
	Up-Q2-4 CogFunc *N* = 37,958,290	Low-Q1 CogFunc *N* = 12,708,756	Up-Q2-4 CogFunc *N* = 1736	Low-Q1 CogFunc *N* = 1,009
Age (years), Mdn (IQR)	66 (63, 72)	74 (67, 80)	67 (63, 73)	71 (65, 80)
PIR, Mdn (IQR)	3.74 (2.02, 5.00)	1.88 (1.14, 3.22)	2.89 (1.48, 4.96)	1.54 (0.99, 2.73)
BMI, Mdn (IQR)	28.2 (24.8, 32.2)	27.4 (24.4, 31.6)	28.2 (24.7, 32.4)	27.8 (24.7, 32.2)
WAIST, Mdn (IQR)	102 (92, 111)	102 (93, 110)	101 (92, 110)	101 (93, 111)
25(OH)D (nmol/L), Mdn (IQR)	80.4 (63.4, 100.0)	76.8 (59.2, 95.7)	76.2 (57.0, 95.7)	71.1 (52.1, 91.3)
25(OH)D2 (nmol/L), Mdn (IQR)	1.45 (1.45, 2.12)	1.45 (1.45, 2.27)	1.45 (1.45, 2.06)	1.45 (1.45, 2.34)
25(OH)D3 (nmol/L), Mdn (IQR)	76.0 (57.3, 94.8)	70.4 (50.0, 90.5)	70.0 (50.0, 90.3)	63.8 (43.9, 83.7)
C3-epi-25(OH)D3 (nmol/L), Mdn (IQR)	4.41 (2.67, 7.14)	3.76 (2.15, 5.72)	3.77 (2.23, 6.18)	3.3 (1.74, 4.99)
PHQ-9, Mdn (IQR)	26 (23, 27)	25 (21, 27)	26 (23, 27)	25 (21, 27)
CERAD total, Mdn (IQR)	28 (25, 32)	19 (16, 22)	28 (25, 32)	20 (16, 23)
Animal fluency, Mdn (IQR)	20 (16, 23)	12 (10, 15)	19 (16, 22)	12 (10, 15)
DSST, Mdn (IQR)	58 (49, 67)	34 (26, 42)	54 (46, 64)	31 (23, 39)
Gender, *n* (%)				
Female	21,218,539 (56)	6,208,069 (49)	963 (55)	443 (44)
Male	16,739,751 (44)	6,500,687 (51)	773 (45)	566 (56)
Marital Status, *n* (%)				
With Partner	25,854,309 (68)	7,026,791 (55)	1,005 (58)	513 (51)
Without Partner	12,097,039 (32)	5,676,734 (45)	273 (16)	264 (26)
Education level, *n* (%)				
Less than 9^th^ grade	686,954 (2)	2,137,769 (17)	53 (3)	248 (25)
9-11th grade	2,842,746 (7)	2,265,988 (18)	173 (10)	205 (20)
High school Grad/GED	7,496,909 (20)	3,700,225 (29)	391 (23)	257 (26)
Some college or AA degree	13,322,250 (35)	2,736,540 (22)	594 (34)	184 (18)
College graduate or above	13,609,431 (36)	1,859,597 (15)	525 (30)	113 (11)
Season of exam, *n* (%)				
November–April	15,079,690 (40)	5,402,868 (43)	771 (44)	470 (47)
May–October	22,878,600 (60)	7,305,888 (57)	965 (56)	539 (53)
Smoking status, *n* (%)				
Current smoker	3,970,262 (10)	1,632,667 (13)	203 (12)	149 (15)
Former smoker	14,664,727 (39)	4,986,823 (39)	650 (37)	385 (38)
Never smoker	19,315,819 (51)	6,083,996 (48)	882 (51)	474 (47)
Alcohol intake, *n* (%)				
1–5 drinks/month	18,333,158 (48)	5,640,719 (45)	841 (48)	480 (48)
5–10 drinks/month	2,145,764 (6)	396,248 (3)	87 (5)	34 (3)
10+ drinks/month	8,251,017 (22)	2,052,142 (16)	310 (18)	126 (13)
Non-drinker	9,204,117 (24)	4,585,532 (36)	497 (29)	365 (36)
Sleep disorder, *n* (%)				
Yes	13,128,159 (35)	4,292,776 (34)	582 (34)	320 (32)
No	24,830,132 (65)	8,415,979 (66)	1,154 (66)	689 (68)
Emphysema, *n* (%)				
Yes	1,635,889 (4)	642,585 (5)	60 (3)	39 (4)
No	36,317,795 (96)	12,046,648 (95)	1,675 (97)	968 (96)
Chronic bronchitis, *n* (%)				
Yes	3,054,402 (8)	758,280 (6)	139 (8)	59 (6)
No	34,805,593 (92)	11,912,827 (94)	1,591 (92)	948 (94)
Heart disease, *n* (%)				
Yes	5,831,794 (15)	3,079,161 (24)	142 (8)	112 (11)
No	32,054,263 (85)	9,584,325 (76)	1,587 (92)	890 (89)
Malignancy, *n* (%)				
Yes	9,185,901 (24)	2,667,362 (21)	364 (21)	171 (17)
No	28,761,699 (76)	10,037,755 (79)	1,370 (79)	837 (83)
Stroke, *n* (%)				
Yes	1,751,782 (5)	1,468,008 (12)	78 (5)	111 (11)
No	36,142,430 (95)	11,240,748 (88)	1,653 (95)	898 (89)
Hyperlipidemia, *n* (%)				
Yes	23,780,451 (63)	8,284,747 (66)	1,087 (63)	640 (64)
No	14,173,232 (37)	4,344,802 (34)	648 (37)	362 (36)
Hypertension, *n* (%)				
Yes	20,890,315 (55)	8,748,225 (69)	1,027 (59)	686 (68)
No	16,976,970 (45)	3,926,637 (31)	707 (41)	320 (32)
Diabetes, *n* (%)				
Yes	7,799,217 (21)	3,873,122 (30)	420 (24)	337 (33)
No	30,154,466 (79)	8,828,019 (70)	1,315 (76)	671 (67)

Up-Q2-4, Upper Quartile 2 to 4; Low-Q1, Lower Quartile 1; CogFunc, cognitive function; Mdn (IQR), median (interquartile range); PIR: poverty income ratio; BMI, body mass index; PHQ-9: depression of patient health questionnaire-9; CERAD, Consortium to Establish a Registry for Alzheimer’s disease; DSST, digit symbol substitution test.

### 3.2 Covariable selection and preliminary analysis for mediation analysis

**Figure 1:**
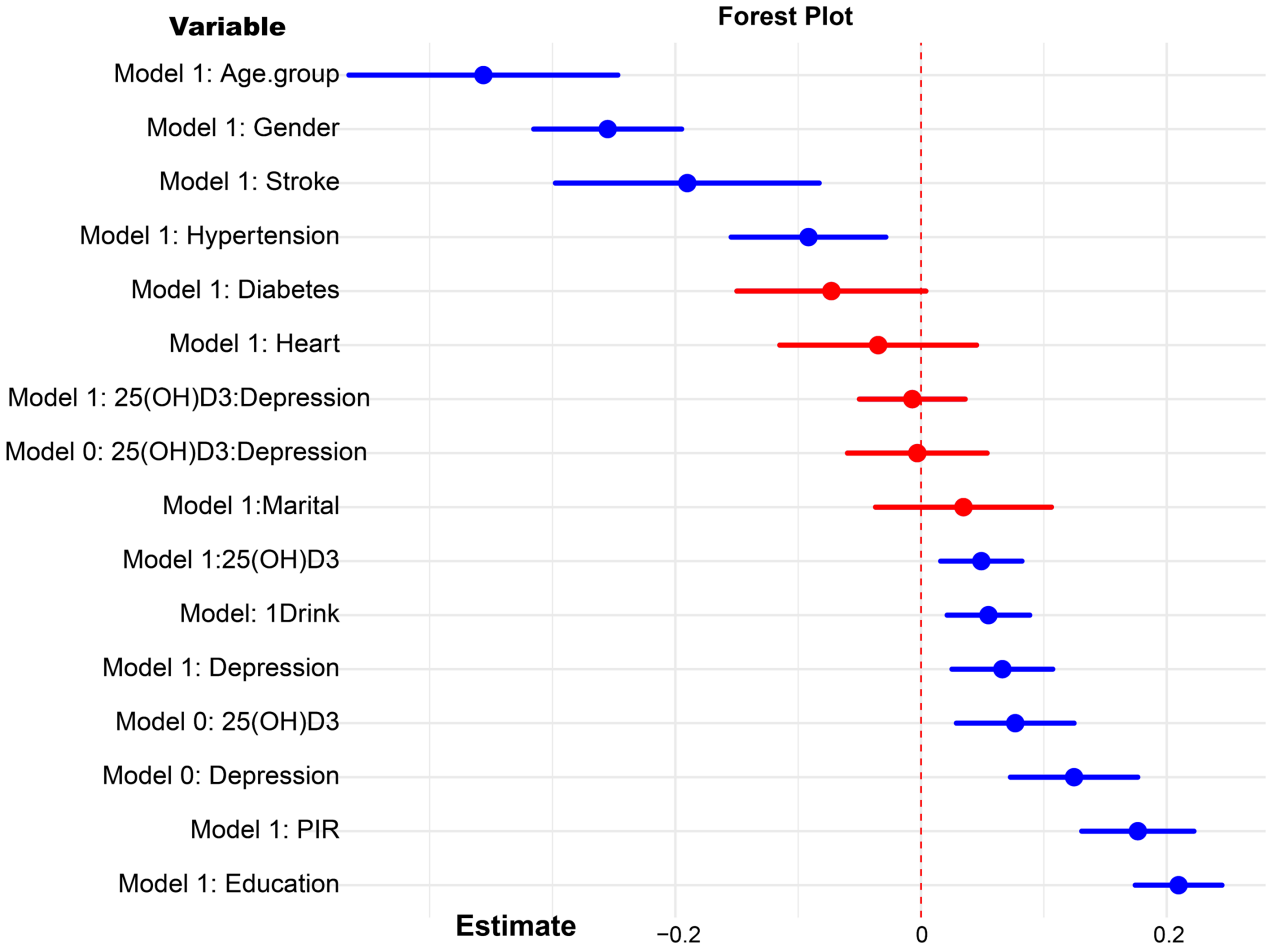
Forest plot for the interaction analysis of 25(OH)D3 and depression on cognitive function. Model 0: crude model, model 1: adjusted for age, gender, education, poverty income ratio (PIR), marital status, drink, stroke, heart disease, diabetes, hypertension.

In our analysis, the covariables selected for further study based on the weighted data were stroke, alcohol consumption, education level, age group, gender, income-to-poverty ratio, diabetes, hypertension, heart disease, and marital status (Supplementary Table S1). Preliminary analysis confirmed the statistical significance of the relationships between 25(OH)D3 levels, depression, and cognitive function, which laid the groundwork for the subsequent mediation analysis. Upon controlling for all selected covariates, both 25(OH)D3 (Estimate = 0.049, *p* = 0.010) and depression (Estimate = 0.066, *p* = 0.005) were identified as independent predictors of cognitive function. A significant positive association was also established between 25(OH)D3 levels and depression scores (Estimate = 0.098, *p* = 0.001). This association persisted (Estimate = 0.094, *p* = 0.001) when taking into account the influence of chronic diseases (stroke, hypertension, diabetes and heart disease). In the results presented in Table 2, the survey-weighted regression analyses demonstrate significant associations between 25(OH)D3 levels, depression scores, and cognitive outcomes across both the entire cohort and most stratified subgroups after adjusting for NHANES complex survey designs. In Figure 1, the analysis reveals no significant interaction between 25(OH)D3 levels and depression scores in influencing cognitive outcomes, both in the univariate model and the multivariate model.

**Table 2:** Survey-weighted regression for independent & mediator variables with outcome.

Cohort	Model 0		Model 1	
	Estimate (95% CI)	*p*	Estimate (95% CI)	*p*
Impact of 25(OH)D3 on cognitive function				
Entire cohort	0.089 (0.044, 0.134)	0.001	0.053 (0.020, 0.086)	0.005
Age < 70	0.168 (0.115, 0.220)	<0.001	0.074 (0.025, 0.123)	0.007
Age ≥ 70	0.071 (0.025, 0.117)	0.005	0.029 (−0.007, 0.065)	0.131
Male	0.105 (0.029, 0.181)	0.011	0.065 (0.003, 0.126)	0.050
Female	0.073 (0.018, 0.127)	0.013	0.045 (0.011, 0.079)	0.016
DM	0.096 (0.034, 0.157)	0.005	0.075 (0.028, 0.122)	0.005
Non-DM	0.076 (0.018, 0.134)	0.016	0.042 (0.002, 0.083)	0.053
HTN	0.073 (0.015, 0.131)	0.020	0.055 (0.013, 0.096)	0.017
Non-HTM	0.119 (0.051, 0.187)	0.002	0.048 (−0.002, 0.099)	0.072
Impact of depression score on cognitive function				
Entire cohort	0.133 (0.083, 0.184)	<0.001	0.070 (0.029, 0.112)	0.001
Age < 70	0.155 (0.093, 0.218)	<0.001	0.060 (0.018, 0.101)	0.011
Age ≥ 70	0.134 (0.084, 0.185)	<0.001	0.084 (0.026, 0.142)	0.009
Male	0.098 (0.044, 0.152)	0.001	0.037 (−0.005, 0.078)	0.095
Female	0.174 (0.109, 0.240)	<0.001	0.088 (0.034, 0.142)	0.004
DM	0.141 (0.066, 0.216)	0.001	0.115 (0.048, 0.183)	0.003
Non-DM	0.115 (0.064, 0.166)	<0.001	0.050 (0.004, 0.096)	0.043
HTN	0.122 (0.056, 0.188)	0.001	0.074 (0.022, 0.126)	0.010
Non-HTM	0.115 (0.044, 0.187)	0.004	0.062 (0.006, 0.119)	0.043

Model 1: crude model; Model 2: adjusted for age, gender, education, poverty income ratio, marital status, drink, stroke, heart disease, DM, HTN. CI, confidence interval; DM, diabetes mellitus; HTN, hypertension.

### 3.3 Selection of stratified weighted mediation analysis models

**Figure 2:**
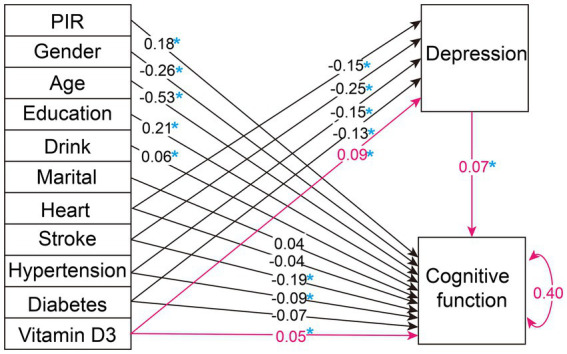
Weighted overall population path analysis diagram. The figure illustrates the path diagram for the variables analyzed in our selected SEM, using the weighted total population. Black lines with associated values represent path estimates on depression and cognitive function, respectively. The red lines highlight the study's focal path estimates. The value of 0.40 adjacent to 'Cognitive function' indicates the estimated residual variance for the dependent variable within the SEM. PIR, poverty income ratio; Heart, heart disease. A blue asterisk indicates statistical significance.

Upon evaluating various fit indices such as the Comparative Fit Index (CFI), Tucker-Lewis Index (TLI), Standardized Root Mean Square Residual (SRMR), Akaike Information Criterion (AIC), Bayesian Information Criterion (BIC), and Sample-Size Adjusted BIC (SABIC), Model 1 proved to be the most appropriate. It demonstrated an optimal balance among these measures, suggesting that it best captures the data structure when compared to other models, as detailed in Table 3. The results of the path analysis for Model 1 in the weighted total population are presented in Figure 2.

**Table 3:** Comparative fit indices for weighted SEM models with varied inclusion of chronic disease covariates.

Model	CFI	TLI	SRMR	AIC	BIC	SABIC
Model 1	0.917	0.682	0.026	12488.72	12612.98	12546.26
Model 2	0.892	0.750	0.028	12524.34	12625.24	12571.22
Model 3	0.874	0.712	0.035	12551.15	12651.75	12597.74
Model 4	0.806	0.466	0.032	13639.77	13704.87	13669.92
Model 5	0.902	0.756	0.041	12587.07	12664.00	12622.70

Model 1: Comprehensive mediation with chronic disease covariates. Model 2: Mediation with chronic disease covariates, selective direct effect without chronic disease covariates. Model 3: Mediation and direct effect without chronic disease covariates. Model 4: Direct effect with all covariates, mediation excluding chronic disease covariates. Model 5: Direct effect with only chronic disease covariates, mediation excluding chronic disease covariates.

CFI, comparative fit index; TLI, Tucker-Lewis index; SRMR, standardized root mean square residual; AIC, akaike information criterion; BIC, bayesian information criterion; SABIC, sample-size adjusted bayesian information criterion.

### 3.4 Mediation analysis for weighted total and subgroup data

Mediation analysis was conducted using Model 1 on both the weighted total population data and weighted subgroup data. As presented in Table 4, significant pathways were identified in the overall data and within the diabetes subgroup. In contrast, other pathways did not show statistical significance either in the mediation effect, direct effect, or total effect.

**Table 4:** Mediation analysis of 25(OH)D3 on cognitive function via depression.

Cohort	Direct effect		Indirect effect		Total effect		Proportion
	Estimate (95% CI)	*p*	Estimate (95% CI)	*p*	Estimate (95% CI)	*p*	Mediated
Entire cohort	0.049 (0.001, 0.096)	0.046	0.006 (0.001, 0.011)	0.014	0.055 (0.007, 0.103)	0.024	11.3%
Age < 70	0.071 (0.004, 0.138)	0.039	0.006 (−0.001, 0.011)	0.059	0.076 (0.010, 0.143)	0.025	7.3%
Age ≥ 70	0.023 (−0.022, 0.068)	0.313	0.006 (0.001, 0.012)	0.040	0.029 (−0.016, 0.074)	0.203	20.5%
Male	0.062 (−0.021, 0.145)	0.144	0.004 (−0.002, 0.010)	0.303	0.066 (−0.017, 0.150)	0.118	6.3%
Female	0.041 (−0.013, 0.095)	0.138	0.007 (0.001, 0.014)	0.027	0.048 (−0.007, 0.103)	0.090	15.0%
DM	0.066 (0.003, 0.130)	0.041	0.013 (0.001, 0.025)	0.031	0.079 (0.013, 0.146)	0.020	16.4%
Non-DM	0.040 (−0.021, 0.100)	0.198	0.004 (−0.001, 0.009)	0.077	0.044 (−0.017, 0.104)	0.155	9.4%
HTN	0.049 (−0.005, 0.103)	0.074	0.008 (0.001, 0.015)	0.039	0.057 (0.003, 0.111)	0.038	13.6%
Non-HTM	0.045 (−0.033, 0.123)	0.255	0.004 (−0.001, 0.009)	0.139	0.049 (−0.029, 0.127)	0.218	7.8%

CI, confidence interval; DM, diabetes mellitus; HTN, hypertension.

### 3.5 Validation of significant pathways

**Figure 3:**
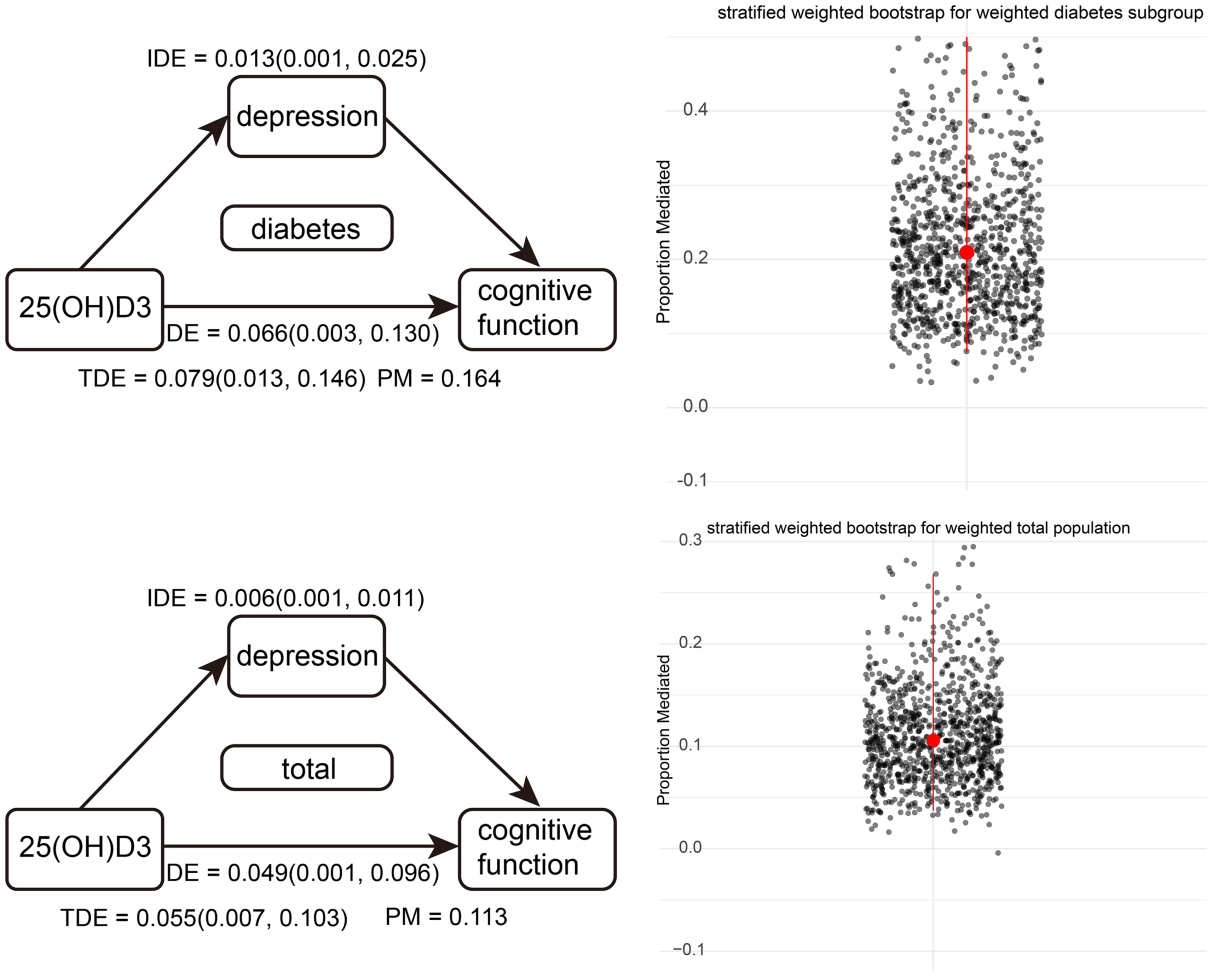
Diagram of weighted overall population and weighted diabetes subgroup pathways, along with stratified weighted bootstrap results. Red dots represent the medians of 1,000 resampling iterations, and red lines indicate confidence intervals.

We developed a stratified weighted bootstrap approach to validate the robustness of mediation pathways in both the weighted overall population and the weighted diabetic subgroup. Figure 3, based on 1,000 bootstrap resamples, illustrates that the mediated effect accounts for 20.9% [0.075, 0.663] in the diabetic subgroup. For the general population, the mediated effect is 10.6% [0.040, 0.268]. Both effects were statistically significant, as indicated by their 95% bootstrap confidence intervals not encompassing zero.

### 3.6 Interactive mediation analysis for depression and diabetes

**Figure 4:**
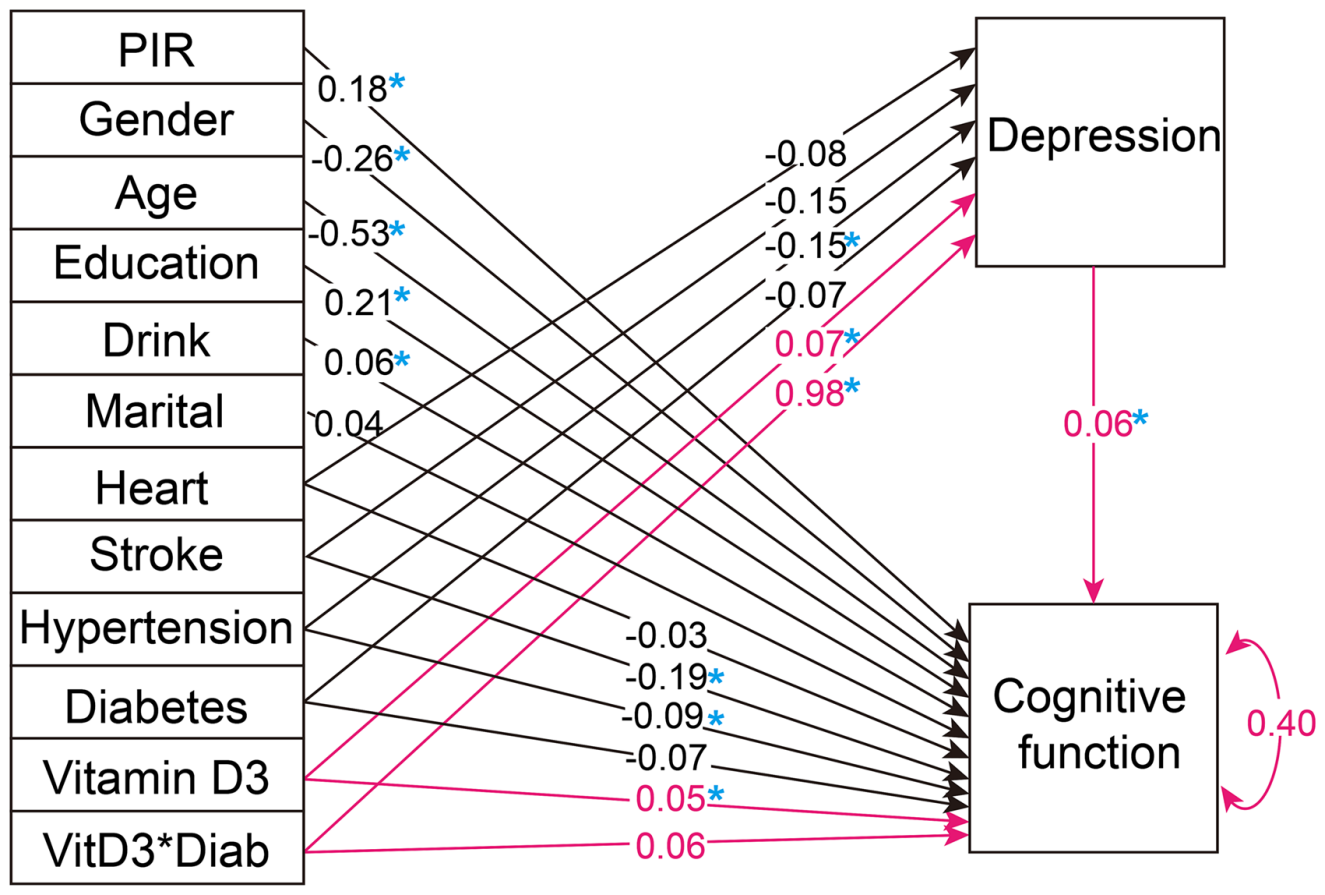
Path analysis diagram for the weighted overall population with the addition of diabetes and depression interaction terms. The figure illustrates the path diagram for the variables analyzed in selected SEM, using the weighted total population. Black lines with associated values represent path estimates on depression and cognitive function, respectively. The red lines highlight the focal path estimates of the mediation effect of depression moderated by diabetes. The value of 0.40 adjacent to 'Cognitive function' indicates the estimated residual variance for the dependent variable within the SEM. PIR, poverty income-ratio; Heart, heart disease. A blue asterisk indicates statistical significance.

We further explored the moderating role of diabetes in the mediation analysis. Figure 4 presents the path analysis results after incorporating interaction terms into both the direct and mediated pathways. Notably, the introduction of an interaction term between diabetes and depression yields a statistically significant estimate of 0.981 in the mediation pathway, surpassing all other variables in the mediation analysis. However, this interaction term is not significant in the direct pathway. Therefore, we consider diabetes to serve as an important moderator of depression in the mediation pathway.

### 3.7 Sensitivity analysis

We applied Model 1 to unweighted data for both overall and subgroup mediation analyses, as shown in Table 4. The results indicate that pathways for the general population and the diabetes subgroup remain significant. Additionally, in the unweighted scenario, numerous other significant pathways emerged, including subgroups of elderly under 70 years, females, non-diabetics, and those with hypertension (Table 5).

**Table 5:** Sensitivity analysis of mediation effects without weighting.

	Direct effect		Indirect effect		Total effect		Proportion
Cohort	Estimate (95% CI)	*p*	Estimate (95% CI)	*p*	Estimate (95% CI)	*p*	Mediated
Entire cohort	0.048 (0.024, 0.074)	<0.001	0.006 (0.003, 0.010)	<0.001	0.055 (0.030, 0.080)	<0.001	11.5%
Age < 70	0.073 (0.039, 0.107)	<0.001	0.005 (0.001, 0.010)	0.022	0.078 (0.044, 0.113)	<0.001	6.6%
Age ≥ 70	0.025 (−0.011, 0.061)	0.169	0.006 (0.001, 0.010)	0.022	0.031 (−0.005, 0.066)	0.094	18.0%
Male	0.071 (0.031, 0.111)	0.001	0.003 (−0.002, 0.009)	0.259	0.074 (0.034, 0.115)	<0.001	4.6%
Female	0.036 (0.004, 0.067)	0.029	0.009 (0.003, 0.015)	0.002	0.045 (0.013, 0.077)	0.006	20.2%
DM	0.059 (0.013, 0.106)	0.012	0.010 (0.001, 0.017)	0.012	0.070 (0.022, 0.115)	0.003	14.9%
Non-DM	0.041 (0.012, 0.071)	0.006	0.005 (0.001, 0.008)	0.015	0.046 (0.016, 0.075)	0.002	10.2%
HTN	0.050 (0.019, 0.081)	0.001	0.009 (0.004, 0.015)	0.001	0.060 (0.029, 0.091)	<0.001	15.9%
Non-HTM	0.043 (0.001, 0.086)	0.049	0.002 (−0.001, 0.006)	0.178	0.046 (0.002, 0.089)	0.040	5.0%

CI, confidence interval; DM, diabetes mellitus; HTN, hypertension.

## 4 Discussion

In our study, leveraging the complex multi-stage sampling design of NHANES and fully accounting for the impact of chronic conditions in the elderly, we conducted an in-depth analysis of how vitamin D affects cognitive function through depression as a mediating factor. Several studies across diverse regions have reported that lower vitamin D is associated with cognitive impairment in the elderly (25, 26), which was consistent with our hypothesis. However, other studies showed that there is no association between vitamin D status and cognitive impairment (27–29). Given the conflicting conclusions in existing research, it’s plausible that the relationship between vitamin D and cognitive function is intricate, potentially influenced by mediating factors and different subgroups. Our research, drawing on weighted data from the entire U.S. civilian, non-institutionalized population, confirms the presence of a pathway in which vitamin D impacts cognitive function through depression, both in the general population and specifically within the diabetic subgroup.

Many NHANES-based studies overlook the importance of the survey’s sampling weights in mediation analyses (30, 31). Building on the R survey package, we integrated it with the complex multi-stage sampling design and combined it with the lavaan package, ensuring that weighted data are utilized throughout the mediation analysis process (32). This methodology not only significantly reduces bias in our estimates but also enhances the statistical power of subgroup analyses by improving generalizability. By considering the design effect and employing appropriate weighting methods, our approach effectively addresses the challenges of unbalanced subgroup sizes, thereby ensuring more accurate and representative analysis outcomes (33). For significant mediation pathways, we implemented a validation process using a robust stratified weighted bootstrap approach, which we developed specifically to accommodate the complex multi-stage sampling design of NHANES. This innovative method underscores the stability and replicability of our findings.

Beyond the general population, the diabetes subgroup also exhibited the significant pathway, prompting further investigation into the mechanisms by which diabetes influences the mediation effect of depression. Our analysis revealed that the interaction term between depression and diabetes presented the highest estimate value (0.981) among all covariates within the mediation pathway, positioning diabetes as a crucial conditional moderator in the mediation of depression. This assertion is well-supported theoretically, as depression and diabetes frequently co-occur (34). Both depression and diabetes are intricately linked to disruptions in brain function and neuroplasticity, evidenced by changes in brain chemistry and structure, such as altered prefrontal neurotransmitter levels and hippocampal atrophy, which impact cognition and mood (35). Research indicates that the prevalence of depression is threefold higher in individuals with prediabetes and twofold higher in diabetes patients compared to the general population (36).

The sensitivity analyses, using unweighted NHANES data across subgroups enhanced our study’s confidence. Beyond the significant pathways identified in the general population and the diabetic subgroup, significant pathways also emerged among the elderly under 70, females, non-diabetics, and hypertensive individuals. This outcome suggests that the impact of vitamin D, mediating through depression on cognitive function, may be more extensive than previously thought, underlining its significance across various population segments. The sensitivity analysis also hints at a potential overestimation of effects when the survey design is not considered, thereby emphasizing the importance of incorporating NHANES weighting throughout our research for accurate and meaningful public health insights. Our findings highlight the potential for interventions such as targeted vitamin D supplementation to not only improve vitamin D levels but also address depression as a key intermediary, thereby enhancing cognitive function. This approach holds particular promise for diabetic and elderly populations, where the interplay of vitamin D, depression, and cognitive decline appears most significant. These findings pave the way for future randomized controlled trials designed to evaluate the significant pathways identified in our research. Such trials are crucial for refining and potentially enhancing current public health guidelines and clinical practices for the elderly, particularly in the context of leveraging vitamin D to mediate the impact of depression on cognitive function.

There are several limitations present in the study. Firstly, although our findings are supported by existing literature suggesting potential pathways between vitamin D, depression, and cognitive function, given the cross-sectional nature of our research, it inherently lacks the temporal data necessary to establish causality or the sequence of occurrence among these variables. Secondly, the study is also geographically limited to the U.S. elderly population, and despite controlling for multiple variables, including some collected through self-reported questionnaires which may introduce bias, unmeasured confounders may still exist. Thirdly, since our analysis methods incorporated weights from NHANES’ complex multi-stage sampling design, many machine learning algorithms do not support analyses with complex weights, leading us to employ path analysis. This analytical approach, being a conventional statistical method, is subject to limitations related to *p*-values, confidence intervals, and statistical power. Fourthly, while this study comprehensively analyzes the mediating role of depression in the relationship between vitamin D levels and cognitive function, it does not directly address factors that might influence vitamin D levels themselves. Future research should explore potential variables that influence vitamin D status, such as BMI, gender, and sun exposure, to expand the research directions and deepen our understanding of the underlying determinants.

## 5 Conclusion

In conclusion, our study utilizes NHANES data to uncover significant pathways by which vitamin D affects cognitive function through depression, with diabetes serving as a pivotal moderator. Throughout the mediation analysis, we employed NHANES’ complex multi-stage sampling design with weighted data and validated the mediation effect proportion using a stratified weighted bootstrap approach. Our results are representative of the entire U.S. civilian, non-institutionalized elderly population. The findings highlight the need to concurrently address vitamin D sufficiency and mental health in developing cognitive health strategies for the elderly, emphasizing the importance of customized interventions for individuals with diabetes.

## Data availability statement

Publicly available datasets were analyzed in this study. This data can be found at: https://www.cdc.gov/nchs/nhanes/index.htm?CDC_AA_refVal=https%3A%2F%2Fwww.cdc.gov%2Fnchs%2Fnhanes.htm.

## Ethics statement

The research adhered to the principles of the Declaration of Helsinki and received approval from the Institutional Review Board at the National Centre for Health Statistics.

## Author contributions

CS: Conceptualization, Data curation, Formal analysis, Investigation, Methodology, Software, Validation, Visualization, Writing – original draft, Writing – review & editing. CZ: Funding acquisition, Supervision, Writing – original draft. XD: Writing – original draft. DL: Funding acquisition, Supervision, Writing – original draft.

## Footnotes

^1^ https://wwwn.cdc.gov/nchs/nhanes/tutorials/weighting.aspx

^2^ https://wwwn.cdc.gov/nchs/nhanes/Default.aspx
