# Supplementary material for: Exploring the role of vitamin D in cognitive function: mediation by depression with diabetes modulation in older U.S. adults, a NHANES weighted analysis
Source: Front Nutr. 2024 Jun 4;11:1356071. doi: 10.3389/fnut.2024.1356071 (PMC11183290; doi:10.3389/fnut.2024.1356071)
Supplement: Supplementary file 1 [file Table_1.DOCX]

| Supplementary Table1: The results of weighted Chi-squared tests for categorical variables and Wilcoxon rank-sum tests for continuous variables | | | | |
| --- | --- | --- | --- | --- |
| Characteristic | Overall, N=50,667,046 | Up-Q2-4 CogFunc  N=37,958,290 | Low-Q1 CogFunc  N=12,708,756 | P-value |
| Age(years), Mdn(IQR) | 68(63, 74) | 66(63, 72) | 74(67, 80) | <0.001 |
| PIR, Mdn(IQR) | 3.13(1.67, 5.00) | 3.74(2.02, 5.00) | 1.88(1.14, 3.22) | <0.001 |
| BMI, Mdn(IQR) | 28.0(24.7, 32.1) | 28.2(24.8, 32.2) | 27.4(24.4, 31.6) | 0.132 |
| WAIST, Mdn(IQR) | 102(92, 111) | 102(92, 111) | 102(93, 110) | 0.753 |
| 25(OH)D(nmol/L), Mdn(IQR) | 79.5(62.5, 98.5) | 80.4(63.4, 100.0) | 76.8(59.2, 95.7) | 0.007 |
| 25(OH)D2(nmol/L), Mdn(IQR) | 1.45(1.45, 2.15) | 1.45(1.45, 2.12) | 1.45(1.45, 2.27) | 0.476 |
| 25(OH)D3(nmol/L), Mdn(IQR) | 74.7(55.3, 93.3) | 76.0(57.3, 94.8) | 70.4(50.0, 90.5) | 0.001 |
| C3-epi-25(OH)D3(nmol/L), Mdn(IQR) | 4.18(2.55, 6.65) | 4.41(2.67, 7.14) | 3.76(2.15, 5.72) | <0.001 |
| PHQ-9, Mdn(IQR) | 26(23, 27) | 26(23, 27) | 25(21, 27) | 0.005 |
| CERAD total, Mdn(IQR) | 27(22, 31) | 28(25, 32) | 19(16, 22) | <0.001 |
| Animal fluency, Mdn(IQR) | 18(14, 21) | 20(16, 23) | 12(10, 15) | <0.001 |
| DSST, Mdn(IQR) | 53(41, 64) | 58(49, 67) | 34(26, 42) | <0.001 |
| Gender, n(%) |  |  |  | 0.016 |
| Female | 27426608(54) | 21218539(56) | 6208069(49) |  |
| Male | 23240438(46) | 16739751(44) | 6500687(51) |  |
| Marital Status, n(%) |  |  |  | <0.001 |
| With Partner | 32881101 (65) | 25854309(68) | 7026791(55) |  |
| Without Partner | 17773773 (35) | 12097039(32) | 5676734(45) |  |
| Education level, n(%) |  |  |  | <0.001 |
| Less than 9^th^ grade | 2824723(6) | 686954(2) | 2137769(17) |  |
| 9-11^th^ grade | 5108735(10) | 2842746(7) | 2265988(18) |  |
| High school Grad/GED | 11197134(22) | 7496909(20) | 3700225(29) |  |
| Some college or AA degree | 16058791(32) | 13322250(35) | 2736540(22) |  |
| College graduate or above | 15469029(31) | 13609431(36) | 1859597(15) |  |
| Season of exam, n(%) |  |  |  | 0.458 |
| November-April | 20482558(40) | 15079690(40) | 5402868(43) |  |
| May-October | 30184488(60) | 22878600(60) | 7305888(57) |  |
| Smoking status, n(%) |  |  |  | 0.276 |
| Current smoker | 5602928(11) | 3970262(10) | 1632667(13) |  |
| Former smoker | 19651549(39) | 14664727(39) | 4986823(39) |  |
| Never smoker | 25399815(50) | 19315819(51) | 6083996(48) |  |
| Alcohol intake, n(%) |  |  |  | <0.001 |
| 1-5 drinks/month | 23973877(47) | 18333158(48) | 5640719(45) |  |
| 5-10 drinks/month | 2542012(5) | 2145764(6) | 396248(3) |  |
| 10+ drinks/month | 10303159(20) | 8251017(22) | 2052142(16) |  |
| Non-drinker | 13789650(27) | 9204117(24) | 4585532(36) |  |
| Sleep disorder, n(%) |  |  |  | 0.767 |
| Yes | 17420935(34) | 13128159(35) | 4292776(34) |  |
| No | 33246111(66) | 24830132(65) | 8415979(66) |  |
| Emphysema, n(%) |  |  |  | 0.563 |
| Yes | 2278473(4) | 1635889(4) | 642585(5) |  |
| No | 48364442(96) | 36317795(96) | 12046648(95) |  |
| Chronic bronchitis, n(%) |  |  |  | 0.090 |
| Yes | 3812682(8) | 3054402(8) | 758280(6) |  |
| No | 46718420(92) | 34805593(92) | 11912827(94) |  |
| Heart disease, n(%) |  |  |  | <0.001 |
| Yes | 8910955 (18) | 5831794(15) | 3079161(24) |  |
| No | 41638589 (82) | 32054263(85) | 9584325(76) |  |
| Malignancy, n(%) |  |  |  | 0.196 |
| Yes | 11853262(23) | 9185901(24) | 2667362(21) |  |
| No | 38799454(77) | 28761699(76) | 10037755(79) |  |
| Stroke, n(%) |  |  |  | <0.001 |
| Yes | 3219790(6) | 1751782(5) | 1468008(12) |  |
| No | 47383177(94) | 36142430(95) | 11240748(88) |  |
| Hyperlipidemia, n(%) |  |  |  | 0.152 |
| Yes | 32065198(63) | 23780451(63) | 8284747(66) |  |
| No | 18518034(37) | 14173232(37) | 4344802(34) |  |
| Hypertension, n(%) |  |  |  | <0.001 |
| Yes | 29638540(59) | 20890315(55) | 8748225(69) |  |
| No | 20903608(41) | 16976970(45) | 3926637(31) |  |
| Diabetes, n(%) |  |  |  |  |
| Yes | 11672339(23) | 7799217(21) | 3873122(30) | 0.003 |
| No | 38982485(77) | 30154466(79) | 8828019(70) |  |
| Abbreviations: Up-Q2-4, Upper Quartile 2 to 4; Low-Q1, Lower Quartile 1; CogFunc, cognitive function; Mdn(IQR), median(interquartile range); PIR: poverty income ratio; BMI, body mass index; PHQ-9: depression of patient health questionnaire-9; CERAD, Consortium to Establish a Registry for Alzheimer’s disease; DSST, digit symbol substitution test | | | | |
