# Supplementary material for: Exploring the role of vitamin D in cognitive function: mediation by depression with diabetes modulation in older U.S. adults, a NHANES weighted analysis
Source: Front Nutr. 2024 Jun 4;11:1356071. doi: 10.3389/fnut.2024.1356071 (PMC11183290; doi:10.3389/fnut.2024.1356071)
Supplement: Supplementary file 2 [file Data_Sheet_1.PDF]

Structural Equation Modeling (SEM) for Investigating the Mediation Effect of Depression on Cognitive Scores

In this study, we employed Structural Equation Modeling (SEM) to explore the mediation effect of depression on the relationship between various health conditions and overall cognitive scores. The SEM framework allows for the inclusion of covariates to adjust for potential confounders in the mediation and direct effects. Specifically, we formulated our model as follows:

Mediation Model Specification

The mediation model incorporates depression as a mediator in the effect of various health conditions (independent variables) on the overall cognitive score (dependent variable). The model also includes covariates to adjust for other factors that might influence the mediator. The model is specified for each health condition (var) as:

$$\text{depression.total} = \alpha \times var + \beta_1 \times \text{Stroke} + \beta_2 \times \text{Diabetes} + \beta_3 \times \text{Hypertension} + \beta_4 \times \text{Heart} + \epsilon_1$$

where:

- α represents the effect of the health condition (var) on depression total score.
- $\beta_1, \beta_2, \beta_3, \beta_4$ are the coefficients for the covariates Stroke, Diabetes, Hypertension, and Heart, respectively.
- ϵ_1 denotes the error term for the mediation equation.

Direct Effect Model Specification

The direct effect model examines the impact of health conditions and the mediator (depression) on the overall cognitive score, adjusting for other covariates:

$$\text{overall\_cognitive\_score} = \gamma \times var + \delta \times \text{depression.total} + \sum_{i=1}^{n} \theta_i \times \text{Covariate}_i + \epsilon_2$$

where:

- γ is the direct effect of the health condition (var) on the overall cognitive score.
- δ represents the effect of depression total score on the overall cognitive score.
- θ_i are the coefficients for each covariate included in the model ($i = 1$ to n; covariates include Stroke, Drink, Education, Age group, Gender, INDFMPIR, Diabetes, Hypertension, Heart, Marital status).
- ϵ_2 is the error term for the direct effect equation.

Indirect and Total Effects

The indirect effect of each health condition (var) on the overall cognitive score through depression is calculated as the product of the coefficients from the mediation and direct effect models:

$$\text{indirect} = \alpha \times \delta$$

The total effect of each health condition on the overall cognitive score is the sum of the direct and indirect effects:

$$\text{total} = \gamma + \text{indirect}$$
