# Supplementary material for: Exploring the role of vitamin D in cognitive function: mediation by depression with diabetes modulation in older U.S. adults, a NHANES weighted analysis
Source: Front Nutr. 2024 Jun 4;11:1356071. doi: 10.3389/fnut.2024.1356071 (PMC11183290; doi:10.3389/fnut.2024.1356071)
Supplement: Supplementary file 3 [file Image_1.pdf]

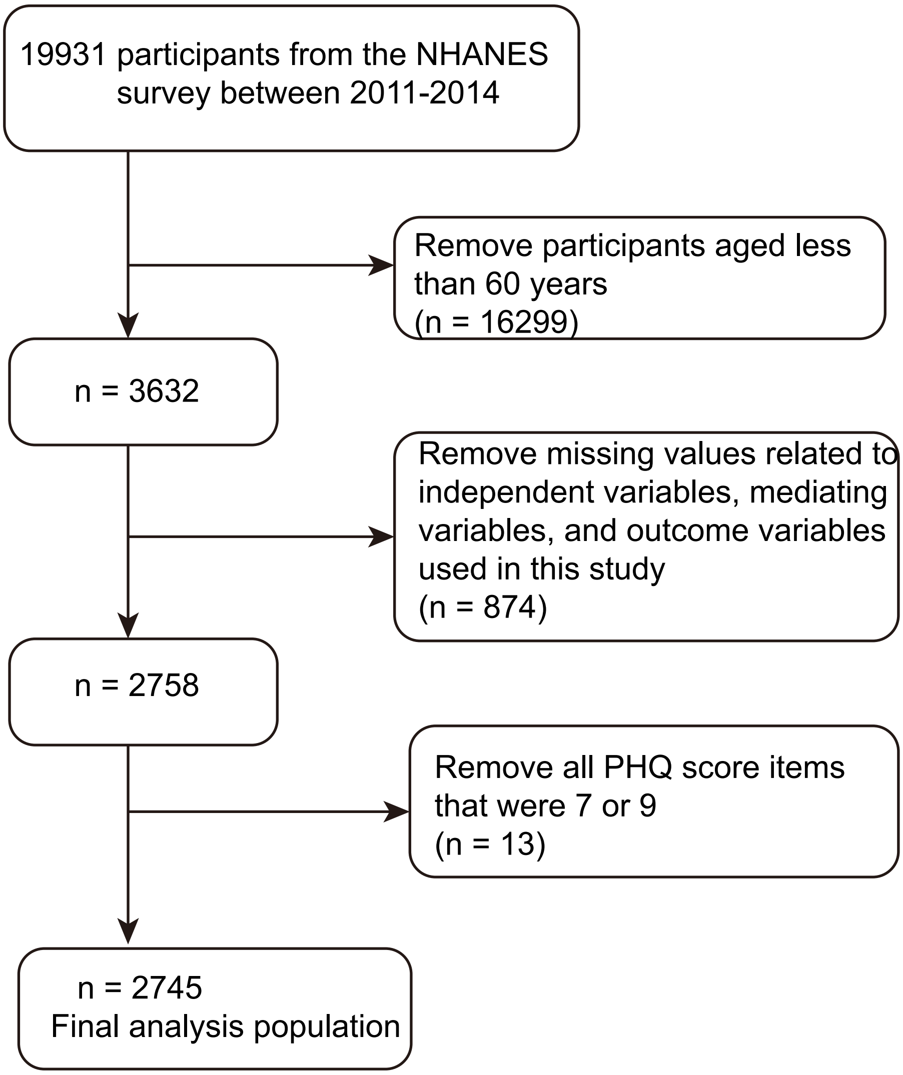

Supplementary Figure 1: Flow chart of the selection process for identifying the eligible population
